# Glucose uptake and distribution across the human skeleton using state-of-the-art total-body PET/CT

**DOI:** 10.1038/s41413-023-00268-7

**Published:** 2023-07-06

**Authors:** Weizhao Lu, Yanhua Duan, Kun Li, Jianfeng Qiu, Zhaoping Cheng

**Affiliations:** 1 Department of Radiology, Shandong First Medical University & Shandong Academy of Medical Sciences, Taian, 271016 China; 2 Department of PET-CT, the First Affiliated Hospital of Shandong First Medical University, Shandong Provincial Qianfoshan Hospital Affiliated with Shandong University, Jinan, 250014 China

**Keywords:** Obesity, Homeostasis, Bone

## Abstract

A growing number of studies have demonstrated that the skeleton is an endocrine organ that is involved in glucose metabolism and plays a significant role in human glucose homeostasis. However, there is still a limited understanding of the in vivo glucose uptake and distribution across the human skeleton. To address this issue, we aimed to elucidate the detailed profile of glucose uptake across the skeleton using a total-body positron emission tomography (PET) scanner. A total of 41 healthy participants were recruited. Two of them received a 1-hour dynamic total-body ^18^F-fluorodeoxyglucose (^18^F-FDG) PET scan, and all of them received a 10-minute static total-body ^18^F-FDG PET scan. The net influx rate (K_i_) and standardized uptake value normalized by lean body mass (SUL) were calculated as indicators of glucose uptake from the dynamic and static PET data, respectively. The results showed that the vertebrae, hip bone and skull had relatively high K_i_ and SUL values compared with metabolic organs such as the liver. Both the K_i_ and SUL were higher in the epiphyseal, metaphyseal and cortical regions of long bones. Moreover, trends associated with age and overweight with glucose uptake (SUL_max_ and SUL_mean_) in bones were uncovered. Overall, these results indicate that the skeleton is a site with significant glucose uptake, and skeletal glucose uptake can be affected by age and dysregulated metabolism.

## Introduction

The human skeleton is a multifunctional organ that undergoes a constant remodeling process through the highly opposing actions of two types of bone cells: osteoclasts and osteoblasts^1^. Osteoclasts are responsible for the resorption of old and damaged bone tissues, and osteoblasts form new bone tissues^1^. The process of bone resorption and bone formation requires a substantial amount of energy, and when the energetic demands are not met, bone remodeling is suppressed^2,3^. Recent studies have suggested that there is cross-talk between bone and glucose homeostasis^2–4^. In common chronic diseases such as diabetes and obesity, the function of bone in the regulation of glucose homeostasis is critical in determining skeletal fragility and osteoporotic fractures^5^. Therefore, it is of clinical significance to investigate glucose metabolism across the human skeleton.

Recent advances have suggested that bone not only serves as a structural scaffold but also as an endocrine organ involved in the regulation of glucose homeostasis^6^. Osteocalcin is a bone matrix protein specifically synthesized and secreted by osteoblasts^7^. Basic animal studies have found that osteocalcin can act on pancreatic β cells to promote insulin secretion and increase insulin sensitivity^7,8^. Osteoclasts also play a critical role in glucose metabolism^9^. In addition to basic research, glucose metabolic imaging is of significant importance because it can detect glucose uptake and its distribution in animals and humans in vivo^10^. Radiolabeled glucose analogs, such as ^18^F-fluorodeoxyglucose (^18^F-FDG), can be used to quantify glucose utilization and uptake via positron emission tomography (PET) imaging^11^. A previous PET imaging study on mice demonstrated that bone accumulated a large portion of the total dose of ^18^F-FDG, and insulin administration further increased the skeletal accumulation of the glucose analog ^12^.

Despite mounting evidence of the cross-talk between bone and glucose homeostasis and existing studies on skeletal glucose uptake using animal models, knowledge of the glucose uptake and distribution across the entire human skeleton is limited. The reasons may be attributed to the low sensitivity and inadequate axial field of view (aFOV) of previous PET scanners in providing whole-body skeletal imaging of glucose metabolism and glucose uptake^13^. The recently developed total-body PET/computed tomography (CT) system by the EXPLORER consortium has an aFOV of 194 cm, allowing for the coverage of the whole human body simultaneously^14,15^. Moreover, uEXPLORER shows a gain in sensitivity of approximately 40 times compared with conventional PET scanners^16^. It was hypothesized that glucose uptake imaging in bone across the human body could be achieved via the uEXPLORER system. Therefore, in this study, a total-body uEXPLORER PET/CT scanner was used to depict the glucose uptake profiles of bones throughout the body. In addition, the effects of age and overweight on the uptake of glucose by the skeleton were investigated.

## Materials and Methods

### Participants

This cross-sectional study was approved by the Ethics Committee of Shandong First Medical University. Informed consent was obtained from all participants. Participants were included based on the following criteria: (1) postprandial glucose (PPG) concentrations between 3.9 mmol·L^−1^ and 7.8 mmol·L^−1^; (2) absence of systemic hypermetabolic lesions such as tumors; (3) absence of metabolic diseases; (4) absence of cardiovascular diseases; and (5) no self-reported significant or unstable medical history. A total of 41 participants (all right-handedness, 17 females and 24 males, aged between 31 and 77 years old) were included in this study. Table 1 provides demographic details for these participants.

**Table 1:** Demographics of the 41 participants

Variable	Value*
Age	55.12 ± 11.81 (31–77)
Sex	24 M/17 F
Body mass/kg	69.32 ± 13.61 (45–100)
Height/m	1.68 ± 0.07 (1.55–1.80)
BMI/(kg·m^−2^)	24.26 ± 3.57 (16.73–30.86)
PPG concentration/(mmol·L^−1^)	5.81 ± 0.82 (4.4–7.6)

^*^Continuous variables are presented as the mean ± standard deviation (minimum value – maximum value)

### Total-body PET/CT acquisition

PET images were collected via the uEXPLORER PET/CT system (United Imaging Healthcare, Shanghai, China) at the First Affiliated Hospital of Shandong First Medical University. Participants were injected with ^18^F-FDG based on their body weight (2.96 MBq·kg^−1^ [0.08 mCi·kg^−1^]) after fasting for at least 6 h. Two female participants (51 and 61 years old) were subjected to a 1 h dynamic PET scan immediately after the injection. The dynamic PET data were reconstructed into a total of 92 volumes, including 30 × 2 s, 12 × 5 s, 6 × 10 s, 4 × 30 s, 25 × 60 s, and 15 × 120 s; voxel size = 1.667 × 1.667 × 2.886 mm^3^, with 2 iterations, 20 subsets, and necessary corrections (including attenuation and scattering corrections). Sixty minutes after injection, a static total-body PET/CT scan was performed for 10 min. Static PET images were reconstructed via time-of-flight and point spread functions, matrix = 192 × 192, slice thickness = 2.886 mm, voxel size = 3.125 × 3.125 × 2.886 mm^3^, with 2 iterations, 20 subsets, Gaussian filter (full width at half maximum = 3 mm), and necessary corrections.

In addition, a total-body high-resolution CT scan was performed with the following parameters: voltage = 120 kVp, matrix = 515 × 512, slice thickness = 1.5 mm, and all CT images were reconstructed with a voxel size of 0.976 6 × 0.976 6 × 1.500 0 mm^3^.

### Image processing

Total-body net influx rate (K_i_) maps were calculated from the 4D dynamic PET data using a 2-tissue-compartment model to fit the dynamic data^17^. In brief, an image-derived input function was extracted from the ascending aorta, and a time delay was taken into consideration during this process with a time delay parameter to be estimated. Finally, the model was optimized using the Levenberg‒Marquardt algorithm to generate the optimal parametric images (K_i_ map) (see Supplementary Method 1 and Fig. S1 for a detailed description)^17^.

The total-body standardized uptake value (SUV) normalized by lean body mass (SUL) images was calculated from the static PET images with the following procedure: (1) calculation of total-body SUV images and (2) calculation of lean body mass according to the equations proposed by Hume (Supplementary Methods 2)^18^. Lean body mass was used to normalize the SUV values to finally yield the total-body SUL images.

For each participant, high-resolution CT images were coregistered to the corresponding total-body PET images using affine transformation. Then, total-body K_i_ images and SUL images of the skeleton were obtained using the bone window of the coregistered CT images.

Due to the inconsistent arm positions of the participants during the PET/CT scan, this study focused on the skull, vertebrae, hip bone, bilateral femur, bilateral tibia and bilateral fibula as regions of interest (ROIs) and did not consider the humerus, radius and ulna. The maximal K_i_ and SUL (K_i max_ and SUL_max_) and mean K_i_ and SUL values (K_i mean_ and SUL_mean_) were calculated for each ROI. In addition, K_i max_, K_i mean_, SUL_max_ and SUL_mean_ were also calculated for the brain, heart, bilateral lungs, stomach, liver, spleen, pancreas, large intestine, small intestine, bilateral kidneys, bilateral thigh muscles, and bilateral calf muscles from the total-body K_i_ and SUL images for reference purposes ^13^.

### Statistical analysis

Since only 2 participants underwent dynamic PET scans, the effects of age and overweight on glucose uptake in human bone were only assessed using the SUL from the static PET data. To investigate the effect of age on glucose uptake in human bone, we divided the 41 participants into adult, middle-aged and senior adult groups. The adult group had an age range of 20–39 years (*n* = 5), the middle-aged group had a range of 40–59 years (*n* = 24), and the senior adult group had an age range of 60 years or older (*n* = 12)^19^. Differences in the SUL_max_ and SUL_mean_ from bone ROIs among the 3 groups were assessed via analysis of variance (ANOVA), with the LSD method for post hoc multiple comparisons. *P* < 0.05 was considered statistically significant. In addition, the associations between age and SUL_max_ or SUL_mean_ of the bone ROIs were evaluated by Pearson correlation or quadratic regression analysis. *P* < 0.05 was considered statistically significant.

To explore the effect of overweight on glucose uptake in bone, we divided the 41 participants into the normal weight and overweight groups by body mass index (BMI). The normal weight group (*n* = 23) had a BMI below 24.9 kg· m^−2^, and the overweight group (*n* = 18) had a BMI of 25 kg· m^−2^ or higher^20^. An independent t-test was used to compare the SUL_max_ and SUL_mean_ from bone ROIs between the two groups. Statistical significance was defined as *P* < 0.05. In addition, the association between BMI and glucose uptake in bone was evaluated by calculating the Pearson correlation between BMI and the SUL_max_ or SUL_mean_ of the bone ROIs. Statistical significance was defined as *P* < 0.05.

## Results

### Glucose uptake and distribution of ^18^F-FDG across the human skeleton

**Fig. 1:**
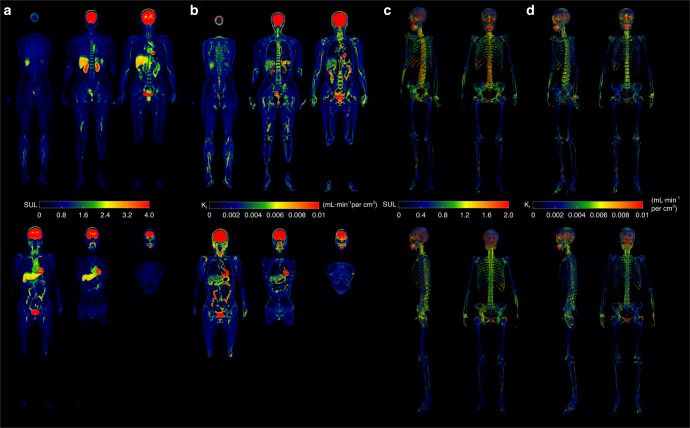
^18^F-FDG uptake profiles across the human body. **a** Total-body SUL map of a 51-year-old female participant. **b** Total-body K_i_ maps of the 51-year-old female participant. The images are displayed in the coronal view. **c** Skeletal SUL maps across the human body of the 51-year-old female participant. **d** Skeletal K_i_ maps across the human body of the 51-year-old female participant. The images are displayed in the oblique, frontal, lateral and back views

Figure 1 shows glucose uptake profiles of the human body, calculated using both dynamic and static PET data. Across the whole body, the highest level of ^18^F-FDG accumulation was observed in the brain, followed by the heart, liver and kidney. The high glucose uptake level in the bladder was attributed to the urinary clearance of ^18^F-FDG (Fig. 1a, b).

Across the skeleton, the vertebrae had the highest glucose uptake level, followed by the skull and hip bone. In contrast, the bones in the four limbs had lower glucose uptake levels. Additionally, in the lower limbs, the epiphyseal and metaphyseal trabecular bone compartments of the femur and tibia exhibited significant glucose uptake, while cortical bone also showed relatively high uptake (Fig. 1c, d).

**Fig. 2:**
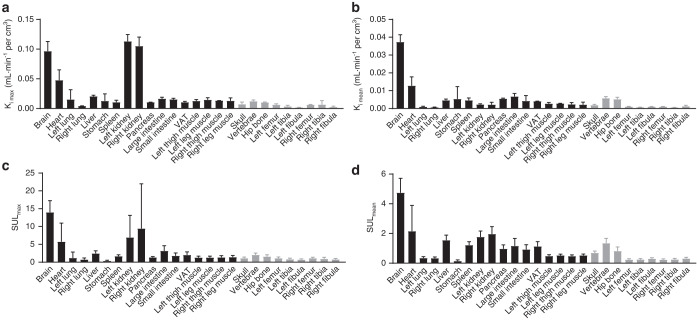
^18^F-FDG uptake profiles of major organs and skeletal ROIs across the whole body. Box plot of the (**a**) K_i max_ and (**b**) K_i mean_ in organs and parts throughout the body for the two female participants. Box plot of the (**c**) SUL_max_ and (**d**) SUL_mean_ in organs and parts throughout the body for all 41 participants. The error bar indicates the mean ± standard deviation

In comparison to the glucose uptake levels in the brain, heart and kidneys, glucose uptake by bones was lower. However, glucose uptake measurements as assessed by both the K_i_ and SUL of the vertebrae were similar to those of the liver and spleen. Glucose uptake measurements as assessed by both the K_i_ and SUL of the hip bone and skull were similar to those of the pancreas, intestines and muscles, indicating that bones play a critical role in the clearance of circulating glucose in the resting state (Fig. 2).

### The association between age and glucose uptake in bone

**Fig. 3:**
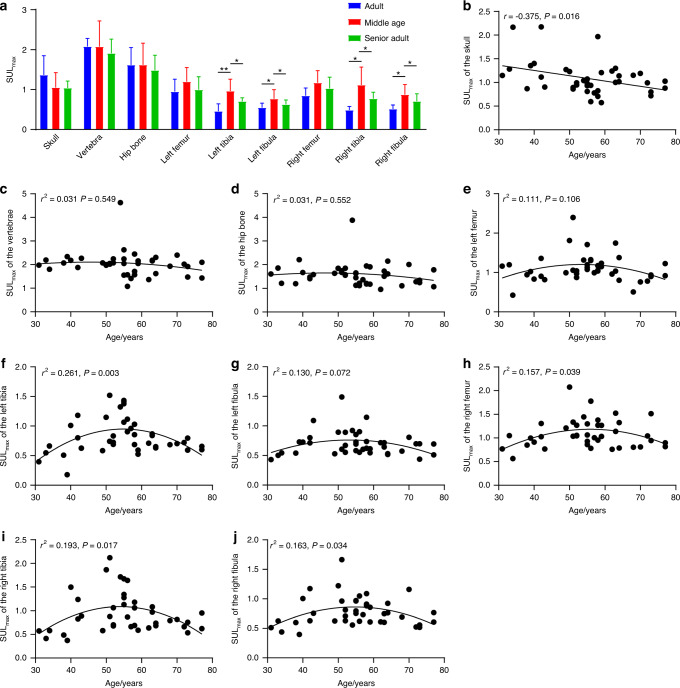
Associations between the SUL_max_ in bone and age across the entire body. **a** Comparisons of the SUL_max_ of the bone ROIs among the three age groups. **P < 0.001, and *P < 0.05 for the post hoc multiple comparison test using the LSD approach. The associations between age and SUL_max_ in the (**b**) skull, (**c**) vertebrae, (**d**) hip bone, (**e**) left femur, (**f**) left tibia, (**g**) left fibula, (**h**) right femur, (**i**) right tibia, and (**j**) right fibula. The curve represents the quadratic fitting curve

To understand the influence of age on glucose uptake in bone, we first divided the 41 enrolled healthy participants into three groups, and the ANOVA results demonstrated that the three groups differed in the SUL_max_ of the bilateral tibia and bilateral fibula (*P* < 0.05) (Fig. 3a). Specifically, the SUL_max_ of the bilateral tibia and bilateral fibula in the adult group (*n* = 5) were significantly lower than those in the middle-aged group (*n* = 24) (*P* < 0.001 for the left tibia, *P* = 0.024 for the left fibula, *P* = 0.001 for the right tibia, and *P* = 0.002 for the right fibula, LSD approach). The SUL_max_ of the bilateral tibia and bilateral fibula in the middle-aged group (*n* = 24) were significantly higher than those in the senior adult group (*n* = 12) (*P* = 0.005 for the left tibia, *P* = 0.044 for the left fibula, *P* = 0.010 for the right tibia, and *P* = 0.040 for the right fibula, LSD approach). Then, we assessed the associations between age and the SUL_max_ of the bone ROIs using Pearson and quadratic regression analyses (Fig. 3b–j). There were significant negative correlations between age and the SUL_max_ of the skull and significant quadratic associations between age and the SUL_max_ of the bilateral tibia, right femur, and right fibula. In addition, quadratic trends between age and the SUL_max_ of the left femur and left fibula were observed, and the associations were significant after outlier removal (Fig. S2).

**Fig. 4:**
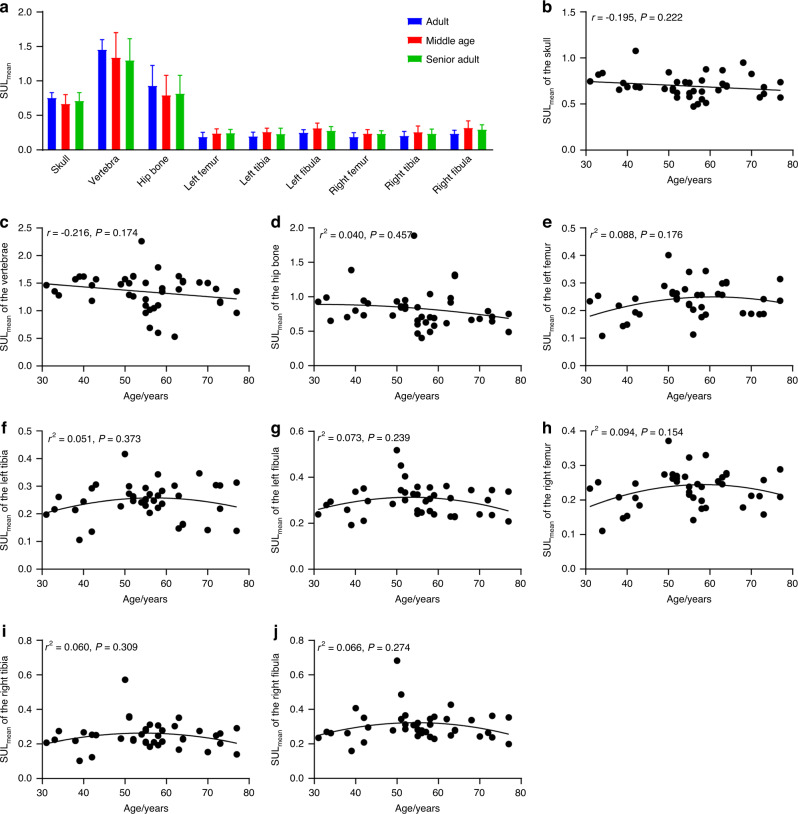
Associations between the SUL_mean_ in bone and age across the entire body. **a** Comparisons of the SUL_mean_ of the bone ROIs among the three age groups. *P < 0.05 for the post hoc multiple comparison test using the LSD approach. The associations between age and SUL_mean_ in the (**b**) skull, (**c**) vertebrae, (**d**) hip bone, (**e**) left femur, (**f**) left tibia, (**g**) left fibula, (**h**) right femur, (**i**) right tibia, and (**j**) right fibula. The curve represents the quadratic fitting curve

The three groups did not differ In the SUL_mean_ of the 9 bone ROIs (Fig. 4a). In terms of associations between age and the SUL_mean_ of bone ROIs, there were also no significant correlations between the SUL_mean_ of each ROI and age (Fig. 4b–j). However, the SUL_mean_ of most bone ROIs demonstrated quadratic trends with age, except for the skull and vertebrae. In particular, after the removal of outliers, the SUL_mean_ of the left femur (*r*^2^ = 0.163, *P* = 0.041), left tibia (*r*^2^ = 0.239, *P* = 0.010), left fibula (*r*^2^ = 0.159, *P* = 0.040), and right femur (r^2^ = 0.179, *P* = 0.035) demonstrated significant quadratic associations with age (Fig. S3).

### The effect of overweight on glucose uptake in bone

**Fig. 5:**
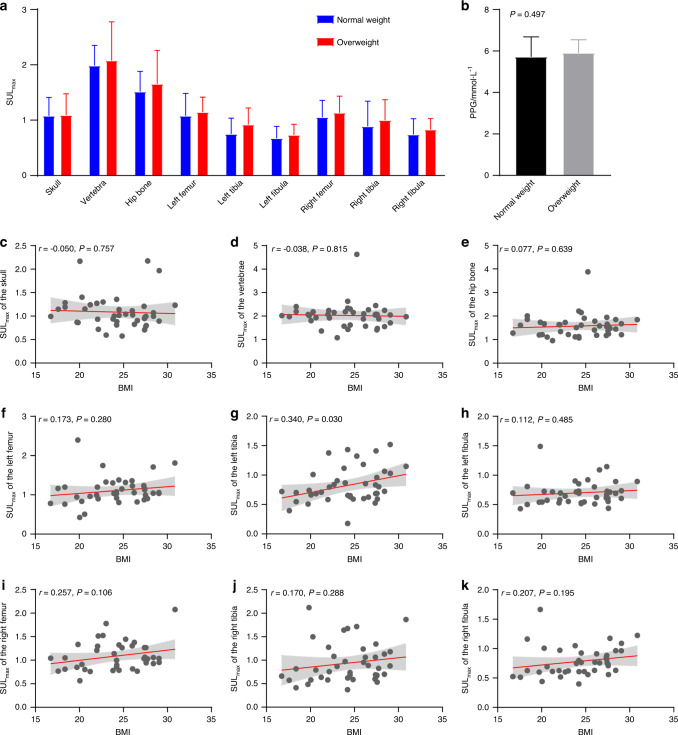
Differences in the SUL_max_ in bone across the human body between normal weight and overweight subjects. **a** Comparisons of the SUL_max_ of the bone ROIs between the normal weight and overweight groups, *P < 0.05. **b** Comparisons of the PPG concentration between the normal weight and overweight groups. The associations between BMI and SUL_max_ in the (**c**) skull, (**d**) vertebrae, (**e**) hip bone, (**f**) left femur, (**g**) left tibia, (**h**) left fibula, (**i**) right femur, (**j**) right tibia, and (**k**) right fibula. The line represents the fitting line, and the filled area represents the 95% confidence interval

Finally, to investigate the effect of overweight on skeletal glucose uptake, we divided the 41 healthy participants into 2 groups according to the BMI threshold of 25.0 kg·m^−2^, and we assessed the differences in the SUL_max_ and SUL_mean_ from the 9 ROIs between the normal weight (*n* = 23) and overweight groups (*n* = 18). As shown in Fig. 5a, the overweight group (*n* = 18) exhibited slightly increased SUL_max_ in the vertebrae, hip bone, and bones of the lower limbs. However, the increases were not significant. Moreover, the two groups did not significantly differ in PPG levels (*P* = 0.497). We further evaluated the associations between BMI and SUL_max_ in bone. As illustrated in Fig. 5c–k, the SUL_max_ of the bones in the lower limb, including the bilateral femur, left tibia and bilateral fibula, demonstrated significantly positive trends with BMI, with the association between BMI and SUL_max_ in the left tibia being statistically significant (Fig. 5g). In addition, the SUL_max_ of the left femur (*r* = 0.366, *P* = 0.020), left fibula (*r* = 0.353, *P* = 0.028), right femur (*r* = 0.338, *P* = 0.035), right tibia (*r* = 0.362, *P* = 0.024), and right fibula (*r* = 0.393, *P* = 0.012) also exhibited significantly positive correlations with BMI after the removal of outliers (Figure S4).

**Fig. 6:**
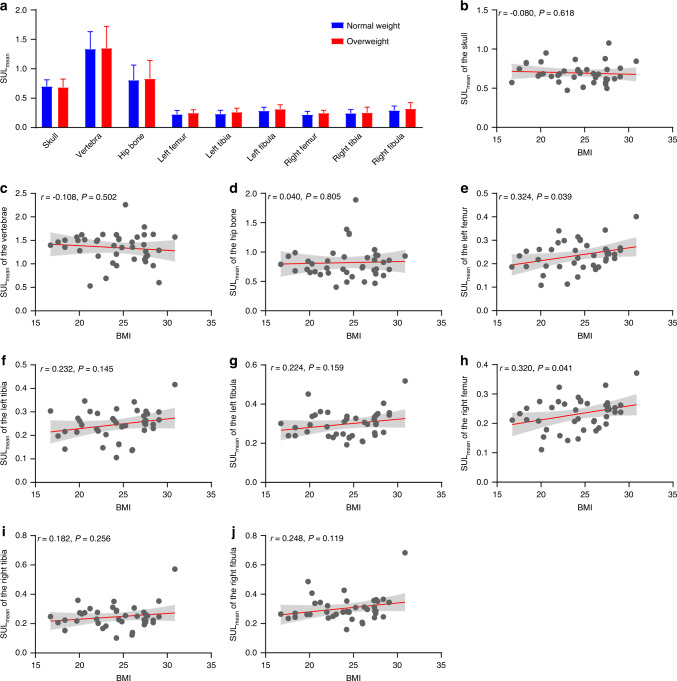
Differences in the SUL_mean_ in bone across the human body between normal weight and overweight subjects. **a** Comparisons of the SUL_mean_ of the bone ROIs between the normal weight and overweight groups, * represents *P* < 0.05. The associations between BMI and SUL_mean_ in the (**b**) skull, (**c**) vertebrae, (**d**) hip bone, (**e**) left femur, (**f**) left tibia, (**g**) left fibula, (**h**) right femur, (**i**) right tibia, and (**j**) right fibula. The line represents the fitting line, and the filled area represents the 95% confidence interval

Similar to the SUL_max_, the overweight group (*n* = 18) exhibited slightly increased SUL_mean_ values in the bones of the lower limbs (Fig. 6a). The SUL_mean_ in the bones of the lower limbs showed positive trends with BMI, with significant associations between BMI and SUL_mean_ of the bilateral femur (Fig. 6e, f). Additionally, the SUL_mean_ of the left tibia and left fibula demonstrated significantly positive correlations with BMI after the removal of outliers (Fig. S5).

## Discussion

The skeleton is an endocrine organ that plays an essential role in regulating human glucose homeostasis. However, glucose uptake and distribution in human bone are not fully understood. In this study, via state-of-the-art total-body PET/CT, we revealed the glucose uptake profiles of bones across the human body. We observed relatively high glucose uptake in the vertebrae, hip bone and skull. We found an effect of age on glucose uptake in bones, which was expressed as inverted U-shaped associations between the SUL_max_ and SUL_mean_ of bone ROIs with age. Moreover, we revealed positive associations between BMI and the glucose uptake profiles of bones.

Over the past decade, studies have gradually uncovered the critical role of bone in human energy metabolism, particularly in the regulation of glucose metabolism. Previous basic studies have revealed the role of both osteoclasts and osteoblasts in glucose homeostasis^4,21–26^. In addition to basic metabolic studies, in vivo visualization of metabolism can be achieved by PET imaging^27^. Several radiotracers, such as ^18^F-FDG and ^11^C-palmitate, have been developed to measure glucose or fatty acid uptake^13,28^. Through ^18^F-FDG PET/CT imaging of mice, Zoch et al. discovered that skeletal accumulation of glucose accounted for a considerable portion of the total dose of ^18^F-FDG and further demonstrated the association between glucose uptake and insulin^12^. Suchacki et al. constructed a skeletal metabolism network via murine ^18^F-FDG PET/CT images and observed tissue interactions beyond skeletal glucose uptake^29^. In humans, skeletal glucose uptake has been used as a biomarker for disease diagnoses^30,31^. Although glucose uptake by the skeletons in mice has been investigated, a study of the glucose uptake and distribution in bone across the entire human body is still lacking, which may be due to the inadequate aFOV and low sensitivity of the existing PET scanners.

The newly developed total-body PET/CT system by the EXPLORER consortium has an aFOV of 194 cm and enables the tracking of metabolic processes throughout the human body simultaneously^14,15^. Additionally, uEXPLORER has a sensitivity that is 40 times higher than that of conventional PET/CT scanners^16^. The ultrahigh sensitivity and extralong aFOV of the total-body PET/CT system allow for total-body skeletal glucose imaging. In this study, via this total-body PET system, we revealed the glucose uptake profiles in the skeleton within the entire human body.

Across the entire human body, glucose uptake varies at different skeletal sites, with the vertebrae having the highest glucose uptake level, followed by the skull and hip bone. The bones in the limbs had relatively low glucose uptake levels. Moreover, glucose uptake by bone, especially the vertebrae and hip bone, was similar to the uptake value by metabolic organs such as the liver and spleen, indicating the critical role of bone in glucose metabolism^1,2^. Bone has been found to affect glucose homeostasis primarily via osteocalcin, insulin and hypoxia signaling^32,33^. Previous studies have demonstrated that insulin receptors have a heterogeneous distribution in bone^34,35^. Bone marrow has been identified as a source of glucose consumption, and evidence suggests that vertebral bone marrow differs from femoral and tibial bone marrow, with the vertebrae consisting of more red marrow and the femur consisting of more bone marrow adipose tissue^36,37^. This difference may account for the variation in the ^18^F-FDG uptake distribution among different skeletal sites. Moreover, glucose uptake by the axial bones, such as the vertebrae and skull, was found to be higher than that of bones in the lower limbs, which was in line with previous studies and was likely because femoral and tibial bone marrow contain more fat and are therefore insulin resistant^37,38^. Additionally, bone formation during remodeling requires energy consumption^39^. The present findings of the higher ^18^F-FDG uptake in the epiphyseal and metaphyseal regions and cortical bone are in accord with the higher numbers of osteoblasts and the higher rate of remodeling at the corresponding skeletal site^12^.

The current findings also suggested that glucose uptake in bones had an inverted U-shaped association with age. In fact, bone turnover and metabolic indicators are related to age^40^. Previous studies have shown that bone mass and bone mineral density begin to decrease between the ages of 30 and 40 years and continue to decline throughout later life^40,41^. However, our results suggested that glucose uptake by the bone began to decline between 50 and 60 years, approximately 20 years after the turnover of bone mass and bone mineral density, which might indicate a compensation mechanism of bone to counteract the loss of mass and density. In addition, a previous animal study suggested that alterations in ^18^F-FDG uptake in bone correspond to decreases in osteoblast numbers^12^. Recently, age-related alterations in osteoblast and osteoclast activity in human bone have been demonstrated^42,43^. However, detailed trajectories between osteogenesis and age are still lacking, which merits further investigation to confirm the results of our study.

The current findings also showed that overweight could affect glucose uptake in bones across the human body. Specifically, as BMI increases, there are increases in glucose uptake in the bilateral femur, bilateral tibia and bilateral fibula. However, the normal weight and overweight groups did not vary with respect to blood glucose levels, indicating that the increased skeletal glucose uptake was not due to insulin sensitivity. Indeed, the relationship between overweight and bone is complex, with both direct and indirect links^44^. Moreover, overweight and obesity have various effects on bone metabolism, including fat accumulation, bone formation, and proinflammatory cytokines^44,45^. The bones may require more energy consumption to maintain the structure and to support the overweight body. Furthermore, the increased glucose uptake in bone in overweight individuals may be indicative of altered cross-talk between bone and glucose homeostasis in these individuals, pointing to skeletal vulnerability associated with overweight and obesity.

There were several limitations that need to be clarified. First, we only selected 9 bone ROIs across the entire human skeleton due to inconsistent arm positions during the PET/CT scan. Future studies should pay attention to other bones across the human body. Second, because the available time of the uEXPLORER was limited, only two participants underwent the 1-hour dynamic PET scan. The overall sample size was also small. The results therefore need to be interpreted with caution. Third, alterations in glucose uptake in bone were not further explored. Further research is warranted to gain a better understanding of the potential biological mechanisms and physiological explanations for the alterations observed in this study.

In conclusion, via state-of-the-art total-body PET/CT, we have elucidated the glucose uptake profiles across the human skeleton in vivo for the first time. Quantitative analysis demonstrated a relatively high level of glucose uptake in bones, especially the vertebrae, hip bone and skull. In addition, the effects of age and overweight on glucose uptake in bone were also revealed. The current study supported the role of the skeleton in the regulation of glucose metabolism and might be helpful in the investigation of skeletal glucose metabolism in relation to age and obesity.

## Supplementary information

Supplementary Materials

## Data Availability

The datasets generated and/or analyzed during the current study are available from the corresponding author on reasonable request.
