# Supplementary material for: Glucose uptake and distribution across the human skeleton using state-of-the-art total-body PET/CT
Source: Bone Res. 2023 Jul 6;11:36. doi: 10.1038/s41413-023-00268-7 (PMC10322940; doi:10.1038/s41413-023-00268-7)
Supplement: Supplementary file 1 — Supplementary Materials [file 41413_2023_268_MOESM1_ESM.docx]

**Supplementary Methods 1. Dynamic PET data processing**

This 2-tissue compartment model is a mathematical model used to describe the dynamic processes of radiotracer uptake, distribution, and elimination in PET imaging. It is based on the assumption that the tissue is composed of two different compartments, an "interstitium" (C_f_(t)) and a "tissue space" (C_m_(t)). The model considers the processes of tracer exchange between these two compartments and the rate of tracer elimination from the body (Supplementary Figure S1). C_p_(t) is the concentration of radiotracer in the plasma, C_f_(t) is the concentration of free radiotracer, and C_m_(t) is the concentration of metabolized tracer in the tissue at time t. The parameter K_1_ is the rate of radiotracer deliver from the plasma to the tissue interstitium, k_2_ is the rate of tracer exiting the tissue space, k_3_ is the rate constant of radiotracer being phosphorylated, k_4_ is the rate of dephosphorylation process, which is neglected in the model. The net influx rate in the unit of mL/min/cm^3^ can be calculated by

$$K_{i}=\frac{K_{1}k_{3}}{k_{2}+k_{3}}$$

The 2-tissue compartment model is able to describe the time-activity curves of PET images and is used to quantify the dynamic processes of radiotracer uptake and metabolism in the body. It is also used to measure the rate of tracer elimination from the body.


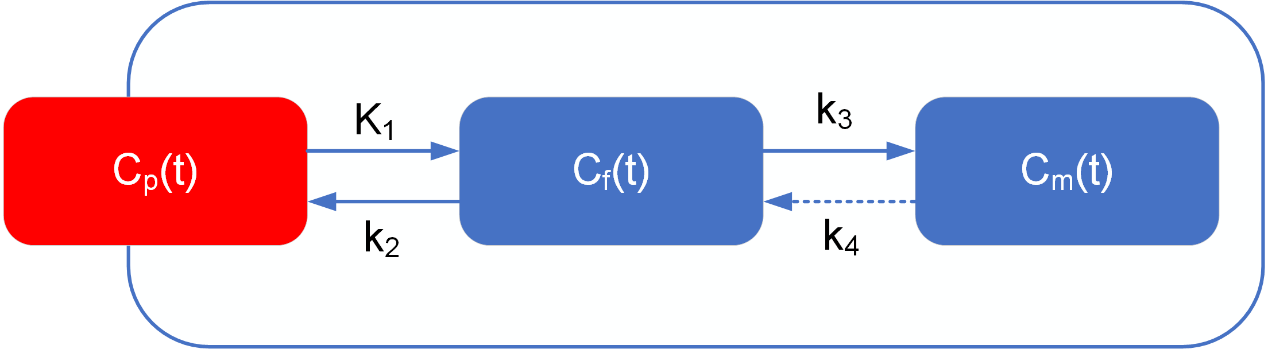


Supplementary Figure S1. Schematic diagram of 2-tissue compartment model.

**Supplementary Methods 2. Estimation of lean body mass**

In this study, lean body mass was calculated according to the equations proposed by Hume [1] as follows:

LBM(M) = 0.32810 × weight + 0.33929 × height − 29.5336 (1)

LBM(W) = 0.29569 × weight + 0.41813 × height − 43.2933 (2)

The unit of weight is kilogram, the unit of height is centimeter. LBM: lean body mass, M: male, W: women

Reference

[1] Hume R. Prediction of lean body mass from height and weight. J Clin Pathol. 1966;19:389-91.


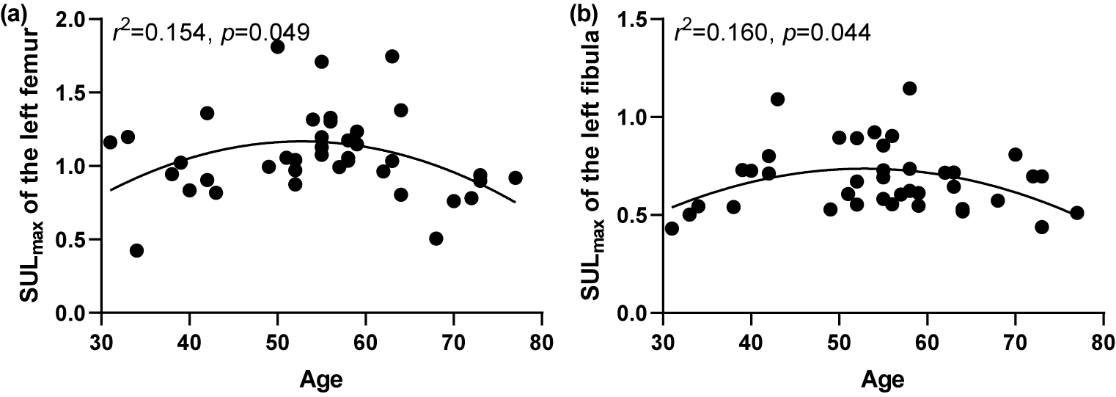


Supplementary Figure S2. Associations between age and SUL_max_ in bone after outlier removal. The association between age and SUL_max_ of the (a) left femur, (b) left fibula. The line represents the quadratic fitting curve.


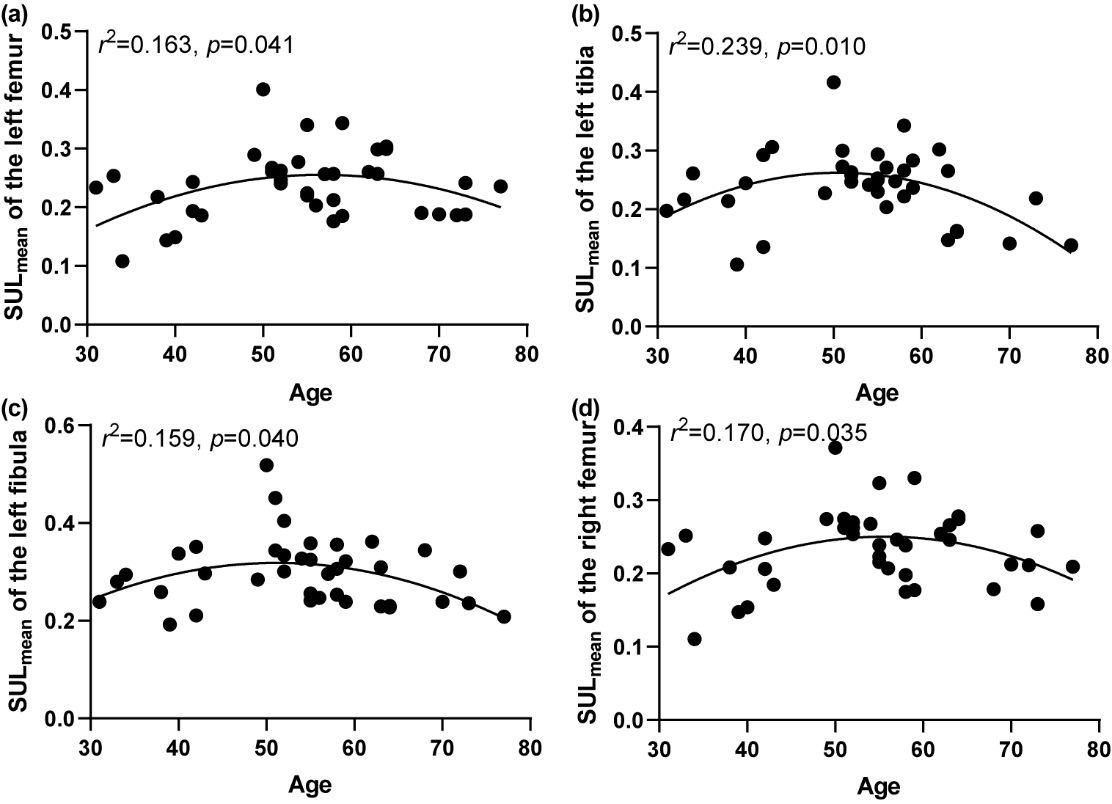


Supplementary Figure S3. Associations between age and SUL_mean_ in bone after outlier removal. The association between age and SUL_mean_ of the (a) left femur, (b) left tibia, (c) left fibula, and (d) right femur. The line represents the quadratic fitting curve.


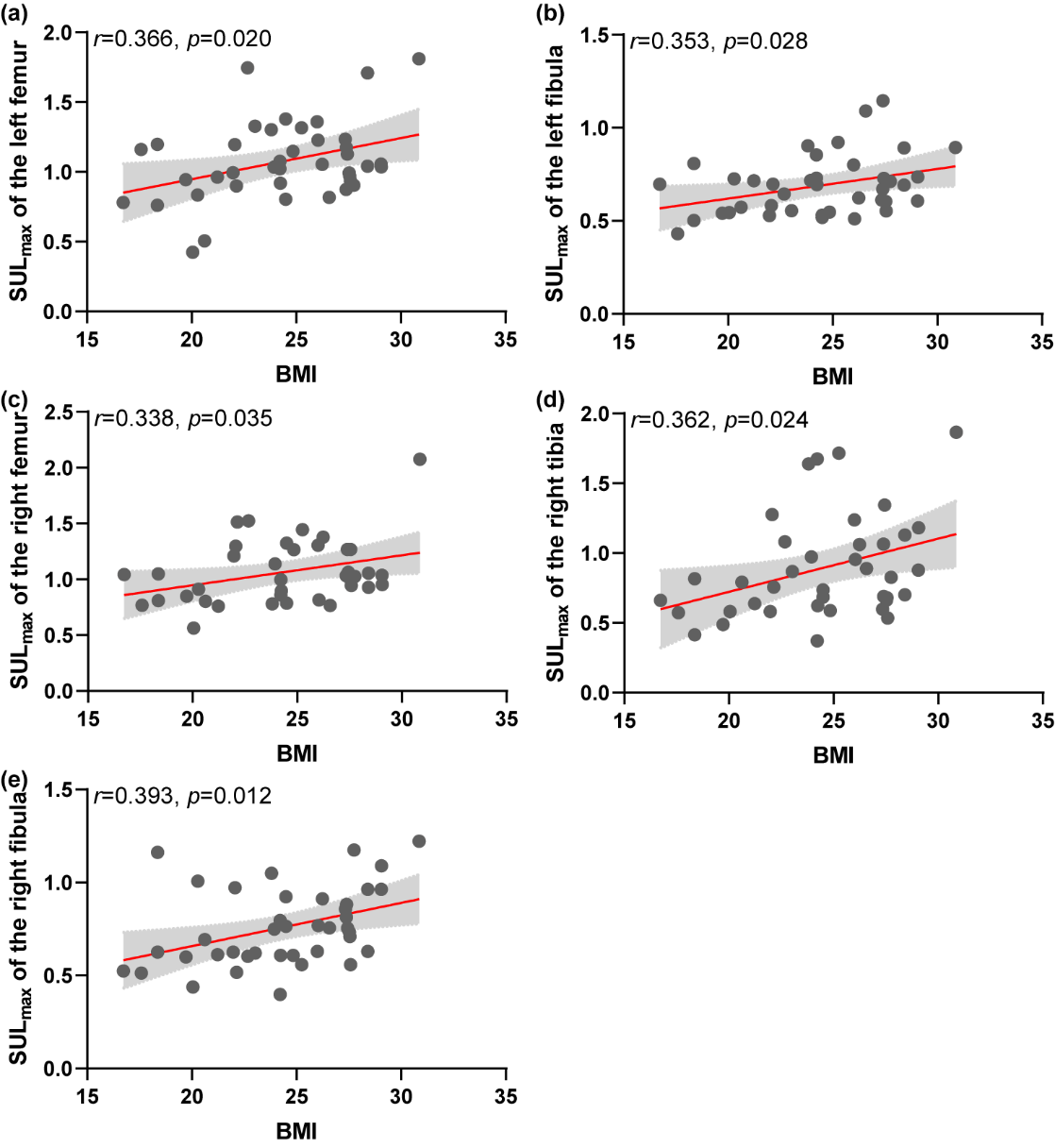


Supplementary Figure S4. Associations between BMI and SUL_max_ in bone after outlier removal. The association between BMI and SUL_max_ of the (a) left femur, (b) left fibula, (c) right femur, (d) right tibia, and (e) right fibula. The line represents the fitting line, and the filled area represents the 95% confidence interval.


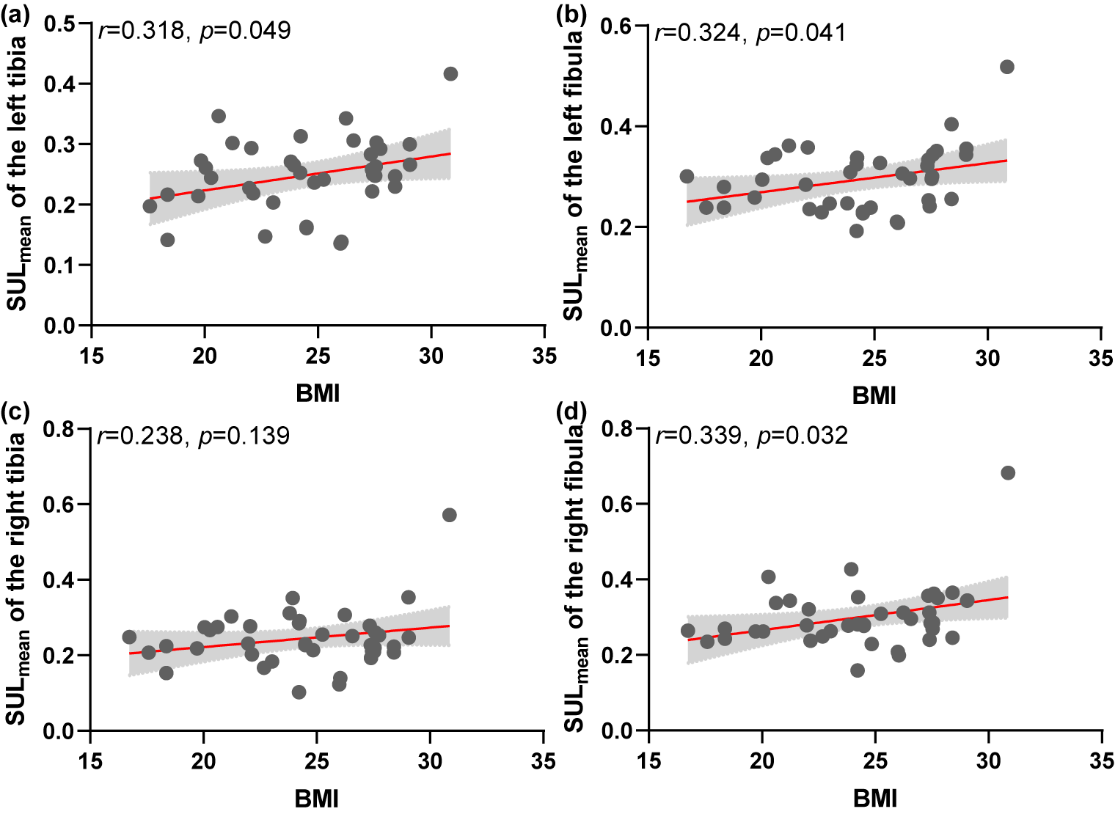


Supplementary Figure S5. Associations between BMI and SUL_mean_ in bone after outlier removal. The association between BMI and SULmean of the (a) left tibia, (b) left fibula, (c) right tibia, and (d) right fibula. The line represents the fitting line, and the filled area represents the 95% confidence interval.
